# Early life stress, low-grade systemic inflammation and weaker suppression of the default mode network (DMN) during face processing in Schizophrenia

**DOI:** 10.1038/s41398-023-02512-4

**Published:** 2023-06-21

**Authors:** Sinead King, David Mothersill, Laurena Holleran, Saahithh Redddi Patlola, Tom Burke, Ross McManus, Marcus Kenyon, Colm McDonald, Brian Hallahan, Aiden Corvin, Derek W. Morris, John P. Kelly, Declan P. McKernan, Gary Donohoe

**Affiliations:** 1 Centre for Neuroimaging, Cognition and Genomics (NICOG), University of Galway, Galway, Ireland; 2 School of Medicine, University of Galway, Galway, Ireland; 3 School of Psychology, University of Galway, Galway, Ireland; 4 Department of Psychology, National College of Ireland, School of Business, Dublin, Ireland; 5 Pharmacology & Therapeutics, University of Galway, Galway, Ireland; 6 Department of Clinical Medicine, Trinity Translational Medicine Institute, Trinity Centre for Health Sciences, St James’s Hospital, Dublin, Ireland; 7 Department of Psychiatry, Trinity Centre for Health Sciences, Trinity College Dublin, Dublin, Ireland; 8 School of Biological and Chemical Sciences, University of Galway, Galway, Ireland

**Keywords:** Molecular neuroscience, Biomarkers

## Abstract

Childhood trauma (CT) is associated with lower cognitive and social cognitive function in schizophrenia. Recent evidence suggests that the relationship between CT and cognition is mediated by both low-grade systemic inflammation and reduced connectivity of the default mode network (DMN) during resting state. This study sought to test whether the same pattern of associations was observed for DMN connectivity during task based activity. Fifty-three individuals with schizophrenia (SZ) or schizoaffective disorder (SZA) and one hundred and seventy six healthy participants were recruited from the Immune Response and Social Cognition (iRELATE) project. A panel of pro-inflammatory markers that included IL-6, IL-8, IL-10, tumour necrosis factor-alpha (TNFa), and C-reactive protein (CRP), were measured in plasma using ELISA. DMN connectivity was measured during an fMRI social cognitive face processing task. Patients showed evidence of low grade systemic inflammation and significantly increased connectivity between the left lateral parietal (LLP) cortex-cerebellum and LLP-left angular gyrus compared to healthy participants. Across the entire sample, IL-6 predicted increased connectivity between LLP-cerebellum, LLP-precuneus, and mPFC-bilateral-precentral-gyri and left postcentral gyrus. In turn, and again in the entire sample, IL-6 (but no other inflammatory marker) mediated the relationship between childhood physical neglect and LLP-cerebellum. Physical neglect scores also significantly predicted the positive association between IL-6 and LLP-precuneus connectivity. This is to our knowledge the first study that provides evidence that higher plasma IL-6 mediates the association between higher childhood neglect and increased DMN connectivity during task based activity. Consistent with our hypothesis, exposure to trauma is associated with weaker suppression of the DMN during a face processing task, and this association was mediated via increased inflammatory response. The findings may represent part of the biological mechanism by which CT and cognitive performance are related.

## Introduction

The default mode network (DMN) is a well-established neural network of functionally and structurally connected brain regions [1] that typically exhibit deactivation during the performance of an externally oriented attention-demanding task [2] and increased activation during a resting state task [3, 4]. DMN functional connectivity (FC) has been largely linked to self-referential thought, mind-wandering, internal-oriented cognition, and social cognitive performance [5]. The biological mechanisms underpinning DMN connectivity changes in response to cognitive stimuli, e.g., from FC during a resting brain state to FC during an externally oriented attention-demanding brain state remains unclear.

Regarding resting state FC (rs-FC), reductions in DMN rs-FC are consistently observed in patients with schizophrenia (SZ) and depression compared to controls [6, 7]. In SZ, recent studies from our group suggest that exposure to childhood trauma (CT) is associated with reduced rs-DMN connectivity [7] and this reduced activation predicted later social cognitive task performance [7].

The biological mechanism for how CT leads to DMN dysconnectivity and social cognitive impairment is unclear. However, we recently demonstrated that the mediating role of rs-DMN between CT and social cognition was itself mediated by higher inflammatory response, measured via the circulating inflammatory cytokine interleukin-6 (IL-6) [8]. In summary, these findings together suggest that individuals with a reported history of CT neglect and evidence of low-grade systemic inflammation (i.e., higher IL-6) are more likely to also exhibit reduced DMN connectivity patterns during rest, in a manner that predicts later behavioural performance of a social cognitive task. Whether the corollary is true - that CT is also associated with a failure to deactivate the DMN during cognitive task performance, and that this failure would be associated with higher inflammatory response (as measured by IL-6) is unknown.

Atypical increased activation/weaker suppression of the DMN during task performance have been reported in some studies of patients with SZ [9–11]. Further to this, as well as the association between IL-6 and resting state DMN FC, there is evidence that IL-6 is also associated with increased activation in multiple regions of the DMN in SZ (Mothersill & King et al., under review). Specifically, a recent fMRI activation study conducted in our lab, found that higher levels of inflammatory cytokine IL-6 predicted increased activation of multiple regions of the DMN during a face processing task (Mothersill & King et al., under review). This finding (when compared to the resting state studies described above i.e. refs. [7, 8]), suggests that the immune response may be associated with weaker suppression of the DMN during a state dependent social cognitive task (i.e., increased activity as opposed to the typical deactivation during tasks). In summary, it is unknown whether IL-6 mediates the association between CT and DMN related social cognitive task performance and if so, whether pro-inflammatory markers other than IL-6 show a similar pattern of associations. Investigating these questions may reveal important insights about the mechanisms of immune relevant DMN dysregulation in response to social cognitive stimuli.

The aim of the present study was to examine the relationship between CT, inflammation and DMN connectivity during performance of a face processing task in patients and healthy participants. Specifically, we sought to [1] examine DMN connectivity differences between both patients with either SZ or schizoaffective disorder (SZA) and healthy participants during a face processing task. Here, we hypothesised that increased connectivity/weaker suppression of DMN would be observed in patients, compared to healthy participants. Next, following on from our resting state DMN findings showing that higher CT predicted reduced DMN rs-FC [7], we sought to investigate whether [2] higher CT was associated with any observed DMN connectivity changes during task performance. Our hypothesis is that CT would predict increased DMN alterations during face processing. Finally, we aimed to investigate whether [3] low-grade systemic inflammation (as measured via a panel of pro- inflammatory markers IL-6, IL-8, IL-10, tumour necrosis factor-alpha (TNFa) and C-reactive protein (CRP)), itself was associated with weaker suppression of the DMN and in turn, whether these markers mediated the association between a past history of CT and DMN dysconnectivity during task performance. We hypothesised that the association between higher CT and higher DMN activation during task performance would be partially mediated by higher levels of IL-6 plasma only i.e., that the association between CT and DMN during social cognition would not be mediated by any other pro-inflammatory markers.

## Methods

### Study participants

Two hundred and twenty-nine participants took part in this study. Fifty three individuals with SZ or SZA were recruited for the Immune Response and Social Cognition (iRELATE) project, as described in our previous studies (King et al., 2021). Briefly, all participants were recruited in Galway and Dublin through community mental health services. All patients had a chronic illness history, a diagnosis of SZ or SZA confirmed using the Structured Clinical Interview for Diagnostic Statistical Manual-IV, and were clinically stable at the time of assessment, as measured using PANSS [12] and HDRS [13]. Exclusion criteria included (i) the presence of a documented history of neurological disorders (e.g., epilepsy), (ii) comorbid axis I mental health disorders, an estimated intelligence quotient (IQ) <70, (iii) a lifetime history of head injury causing loss of consciousness for >1 min, (iv) evidence of substance use disorder within the past 6 months, (v) reported pregnancy or lactation, (vi) contra- indication for MRI scanning (e.g., metal implants or claustrophobia), (vii) the presence of chronic inflammatory illness and (viii) use non-steroidal anti-inflammatory drugs (NSAIDs) in the past 24 h.

In addition, one hundred and seventy six healthy participants were recruited via local and national media advertising in the same regions of Galway and Dublin. Healthy participants were included if they met the criteria of having no documented lifetime personal history of axis I mental health disorder or substance use disorder in the last 6 months, or a first-degree relative with a psychotic disorder, or substance abuse in the last 6 months (based on self-report). Healthy participants also met the same exclusion criteria as the patients described above (i)–(viii).

All participants provided written informed consent in accordance with the guidelines of the local Ethics Committees of the Galway University Hospitals, National University of Ireland Galway and Tallaght Hospital.

### Data collection

#### Plasma isolation and whole blood culture

Plasma isolation and analysis was carried out as described in (King et al. 2021). Briefly, blood samples were taken at approximately the same time of day (9.30 am) from each participant in a 6 ml EDTA tube (BD367873). 2 mL was removed for Toll-like receptor agonist stimulation and analysis (see King, et al., 2021). The remaining sample was centrifuged at 1,200 g for 10 min at ambient temperature. Following centrifugation, plasma was aspirated and stored in 1.5 ml Eppendorf tubes at −80 °C until further analysis. Basal plasma levels of C-reactive protein (CRP), IL-6, IL-8, TNFa and IL-10 itself were measured using a quantikine high sensitivity enzyme-linked immunosorbent assay (ELISA) (Bio-Techne Catalogue Number HS600C) which has an assay sensitivity of 0.09 pg/mL and range of 0.156–10 pg/mL, and read at 450 nm.

#### Childhood trauma

CT was retrospectively assessed using the Childhood Trauma Questionnaire (CTQ)—Short Form [14] a widely used self-report questionnaire comprising 5 subscales of physical abuse, physical neglect, emotional abuse, emotional neglect and sexual abuse. Each subscale includes 5 items, and individuals are requested to answer whether they had experienced the event on a Likert scale ranging from “1” for “never true” to “5” for “very often true.” Following on from our earlier behavioural studies [7, 15] physical neglect CTQ scores—employed as a continuous variable—was used to index childhood trauma in this study.

#### Neuroimaging data acquisition

Brain imaging was carried out on a 3 Tesla Philips Achieva MR system (Philips Medical Systems, Best, The Netherlands) equipped with gradient strength 80 mT/m and slew rate 200 T/m/s using an 8-channel receive-only head coil at the Centre for Advanced Medical Imaging, St. James’s Hospital, Dublin, Ireland.

##### Structural magnetic resonance imaging

High resolution T1-weighted images were obtained using a 3D magnetisation-prepared rapid acquisition with gradient echo (MPRAGE) sequence. The following parameters were used: FOV = 240 × 220 × 162 mm^3^, spatial resolution 0.83 × 0.83 × 0.9 mm3, TR/TE = 6.7/3.1 ms, TI = 808.239929 ms, flip angle = 8°, acquisition time = 5 min 18.8 s.

##### Resting-state functional magnetic resonance imaging during the face processing task

Functional MRI data were acquired during the Faces task using a SE-EPI sequence with a dynamic scan time of 2 s, with: FOV = 240 × 240 × 131 mm, spatial resolution = 3 × 3 × 3.2 mm, 37 slices with interslice gap = 0.349999905 mm, TR/TE = 2000/28 ms, SENSE factor = 2, with SPIR fat suppression and dynamic stabilisation, and flip angle = 90°. For the Faces task, 174 volumes were acquired, taking 5 min and 55.9 s.

#### Neuroimaging data analysis

##### Pre-processing

Images were pre-processed in SPM12 (developed by the Wellcome Department of Cognitive Neurology, Institute of Neurology, London, UK, https://www.fil.ion.ucl.ac.uk/spm/software/spm12/) and MATLAB R2019b 64-bit (v9.7.0.1296695). Functional MRI spatial pre-processing included the following steps [1] Realignment Estimate and Re-slice: Functional data were realigned using SPM12 [2] Co-registration between the T1 image and the re-sliced mean functional image [3] Segmentation and normalization: Functional and anatomical data were normalized into standard MNI space and segmented into grey matter (GM), white matter (WM), and CSF (CSF) tissue classes using SPM12 unified segmentation and normalization procedure [16], and [4] Smoothing of the normalised functional images with an 8 mm full-width half maximum (FWHM) Gaussian kernel. To limit the effects of head motion, the fMRI-faces task scans also underwent “motion scrubbing.” Temporal misalignment between different slices of the functional data, introduced by the sequential nature of the fMRI acquisition protocol, was corrected using the SPM12 slice-timing correction (STC) procedure [17]. Regressors corresponding to the 6 motion correction parameters and their first temporal derivatives (including GM, WM, and CSF) were included to remove variance related to head motion with functional data band-pass filtered (0.01–0.10 Hz).

##### Functional connectivity analysis

Seed-based functional connectivity was run in CONN-fMRI Functional Connectivity toolbox (version 20b) to assess functional connectivity of 4 a priori seeds of the DMN, i.e., the medial PFC, right LP, left LP, and PCC according to the Harvard-Oxford Cortical and Subcortical Atlas (http://www.cma.mgh.harvard.edu/fsl_atlas.html) as implemented in CONN. To test the differences in connectivity between patients and healthy participants, CONN analysis was performed to statistically compare differences between the four DMN seed and connectivity with the rest of the brain. To test the individual effects of CT and higher IL-6 on functional connectivity of the DMN, a series of regression analyses was carried out across all participants. Results were thresholded at *P*_FWE_ < 0.0125 for both the cluster-level and height threshold to account for multiple comparisons.

##### Paus Face recognition test

During the structural and functional MRI session, participants completed the functional social cognitive task called the Paus face Recognition Test [18], which we have previously used to examine face processing in SZ [19]. There were 19 blocks, each of which lasted 18 s and included 4 to 7 videos: nine blocks of circles, five blocks of neutral faces and five blocks of angry faces. In other words, 162 s of the task were circles, while 180 s of the task was faces (i.e., for the majority of the scan, participants were looking at a face).

##### Statistical analysis of demographic and cognitive variables

All statistical analyses were performed using Statistical Package for Social Sciences Version 25.0 (SPSS Inc., IBM). Group comparisons for age, sex, level of education, IQ, CTQ, and IL-6 were assessed with 2- tailed independent samples *t*-tests and Chi-square (*χ*^2^) tests, where appropriate. Significant associations observed, in our initial group comparison analyses, as well as from our regression analyses (i.e., between IL-6 and DMN) within CONN, were subsequently extracted as individual functional connectivity coefficients and imported into SPSS. In order to investigate the relationship between childhood trauma, inflammation, and the significant DMN seeds, we firstly ran a mediation analysis where each individual inflammatory marker was in turn included as a mediating variable (M), CTQ-physical neglect as the independent variable

(IV) and the relevant DMN correlation coefficients as our dependent variable (DV). More specifically, we first ran a mediation analysis where IL-6 was our mediating variable, CTQ-physical neglect was our independent variable and DMN was our dependent variable. We then re-ran the analysis replacing IL-6 with each of the remaining inflammatory markers in turn. For the resulting significant IL-6-DMN coefficients, i.e., where similar mediation analyses were not possible, regression analyses were instead performed to assess if CTQ-neglect predicted variation in the significant IL-6- DMN associations observed. False Discovery rate (FDR) analysis was carried out on all bivariate analyses to account for multiple comparisons.

## Results

### Demographics

Two hundred and twenty nine participants took part in this study. Demographic and clinical characteristics of the study participants are presented in Table 1. Significant differences between patients and healthy participants were observed for age (*t* = 3.5, *p* = 0.001), but not sex (*x* = 2.8, *p* = 0.214). As expected, patients had significantly lower total years of education and IQ compared to healthy participants (*t* = −4.1, *p* = < 0.001; *t* = −7.1, *p* = 0.001 respectively). Patients scored significantly higher on three measures of the childhood trauma (CTQ) questionnaire; i.e., on total CTQ scores (*t* = 1.9, *p* = 0.04), CTQ-sexual abuse scores (*t* = 2.2, *p* = 0.026), and CTQ-physical neglect scores (*t* = 2.3, *p* = 0.02) compared to healthy participants. Higher plasma levels of immune markers C-reactive protein (*t* = 3.3, *p* = .001), IL-6 (*t* = 2.1, *p* = .035), IL-8 (*t* = 2.2, *p* = 0.028), and IL-10 (*t* = 2.3, *p* = 0.029) were observed in patients compared to healthy participants.

**Table 1:** Demographic, clinical, environmental and inflammatory data.

Total Sample	Healthy participants (176)					Patients (53)	
	M (SD)/%	M/%	SD	M/%	SD	*T*-value/χ²	*P*-value
*Sex (female)*	33%	33%		30%		2.8	0.21
*Age*	37 (12.1)	36	12.2	42	11.2	3.5	0.001
*Years of education*	15.1	16.7	3.9	14.9	3.2	−4.1	0.001
BMI*	25.1 (4.8)	24.5	3.8	29.6	5.8	6.9	0.001
IQ*	108.1 (18.9)	113.1	15.8	93.2	17	−7.3	0.001
**Clinical**							
PANSS*		…..		38.5	8.7	…..	…..
HAM-D*	3.2 (3.6)	2.9	3.41	4.8	4.7	3.6	0.001
**Childhood Trauma Questionnaire (CTQ)**							
*CTQ Total Score*	37.6. (12.6)	36.6	12.6	41.1	15.5	1.9	0.04
*Emotional abuse*	8.8 (4.5)	8.5	4.3	9.4	4.9	1.2	0.22
*Emotional neglect*	9.5 (4.5)	9.4	4.4	9.9	4.7	0.71	0.52
*Sexual abuse*	5.8 (2.7)	5.4	1.6	6.9	4.7	2.2	0.02
*Physical abuse*	6.6 (3.0)	6.4	2.7	6.9	3.6	0.85	0.49
*Physical neglect*	6.9 (2.7)	6.4	2.4	7.6	3.3	2.3	0.02
**Inflammatory marker**							
*IL-6 Plasma*	1.9 (3.2)	1.5	1.5	3.3	6.1	2.1	0.03
*C-reactive Protein (CRP)*	2.3 (3.4)	1.8	2.9	4.1	4.4	3.3	0.001
*TNFa plasma*	0.98 (0.41)	0.95	0.38	1.1	47	1.6	0.10
*IL-8 plasma*	3.9 (2.6)	3.7	2.3	4.8	3.3	2.2	0.02
*IL-10 plasma*	0.23 (0.28)	0.21	0.27	0.32	0.27	2.2	0.02

*BMI Boddy-mass Index, *IQ* Intelligence Quotient, *PANSS* Postivie and Negative Symptoms Scale, *HAM-D* Hamilton Depression Severity Inventory, *IL-6* Interleukin-6, *TNFa* Tumour Necrosis Factor alpha

#### Patients exhibit weaker suppression of the DMN compared to healthy participants

**Fig. 1:**
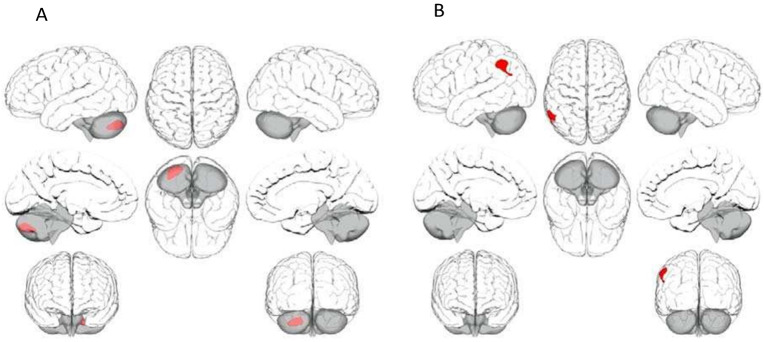
Differences in DMN connectivity between patients and controls. **A**. Patients > healthy participants (LLP seed, cluster 1). **B** Patients > healthy participants (LLP seed, cluster 2).

Given that DMN connectivity was significantly reduced in patients compared to controls during rest conditions [7], we tested whether differences in DMN connectivity were observed between these groups in the same sample, during a face processing task. During faces task performance, patients showed significantly weaker suppression of DMN connectivity compared to healthy participants between the LLP seed and a cluster encompassing the cerebellum. See Table 2 and Fig. 1A. Patients again showed weaker suppression between the LLP and a cluster encompassing the left angular gyrus, the left lateral Occipital Cortex, superior division and the left supramarginal Gyrus, posterior division. See Table 2 and Fig. 1B. No other significant differences were observed.

**Table 2:** Differences in DMN connectivity during a face processing task (patients > healthy participants).

Brain Area	MNI				
	*x*	*y*	z	Voxels	*P*-value
**Medial PFC seed (patients** **>** **healthy participants)**					
No significant clusters					
**L lateral parietal seed—cluster 1 (patients** **>** **healthy participants)**					
470 voxels (80%) covering 24% Cerebelum Crus2 Left 65 voxels (11%) covering 12% Cerebelum 7b Left 6 voxels (1%) covering 0% Cerebelum 8 Left 49 voxels (8%) covering 0% of atlas.not-labelled	−24	−80	−42	590	
					<0.0125 (FWE)
**L lateral parietal seed—cluster 2 (patients** **>** **healthy participants)**					
207 voxels (38%) covering 22% Angular Gyrus Left 160 voxels (29%) covering 3% Lateral Occipital Cortex, superior division Left 132 voxels (24%) covering 12% Supramarginal Gyrus, posterior division Left 52 voxels (9%) covering 0% of atlas.not-labelled	−56	−54	44	551	<0.0125 (FWE)
**R lateral parietal seed (patients** **>** **healthy participants)**					
No significant clusters					
**Posterior cingulate cortex seed (patients** **>** **healthy participants)**					
No significant clusters

#### Childhood trauma predicts weaker suppression of the DMN

Given the evidence reported in our recent paper that childhood trauma predicted reduced connectivity of the DMN during rest, we next tested the corollary hypothesis, that measures of childhood trauma would also predict weaker suppression of the DMN during face processing. The CTQ physical neglect subscale score was used initially to index childhood trauma in this analysis, on the basis that this variable showed previous evidence in the same sample of being associated with both plasma IL-6 and reduced connectivity within the DMN at rest [8]. We found that physical neglect predicted weaker suppression of the DMN, specifically between the LLP seed and the cluster encompassing the cerebellum (*r* = 0.231, *p* = 0.012).

#### Immune response as a mediator of the association between CTQ-neglect and weaker suppression of the DMN during face processing

We next tested whether IL-6 mediated the association between higher CTQ-physical neglect and weaker suppression of the DMN. Mediation analyses, based on 5000 bootstrapped samples indicated that after correcting for age, BMI and IQ, higher IL-6 mediated the association between higher CTQ- physical neglect and weaker suppression of the [1] LLP-cerebellum (βINDIRECT = 0.0179, CI: .0007 to.0422). and [2] LLP-precuneus (βINDIRECT = 0.0179, CI: .0005 to .0053). No other inflammatory marker i.e., CRP, IL- 8, IL-10, or TNFa, significantly mediated the association between CTQ-physical neglect and weaker suppression of the DMN.

#### Higher IL-6 plasma and weaker suppression of the DMN

Because IL-6 was the only significant mediator from the mediation analysis observed above, we next examined the direct relationship between IL-6 and task based DMN connectivity, within CONN. More specifically, regression analyses carried out within CONN were used to investigate whether higher IL- 6 predicted changes in seed-based functional connectivity between each of the 4 DMN seed regions (medial PFC, right LP, left LP, and the PCC) and the rest of the brain. For the medial PFC seed, we found a significant association between IL-6 and weaker suppression of the DMN between the mPFC and a cluster encompassing bilateral precentral gyri and the left postcentral gyrus across all participants. Results were thresholded at the cluster-level correction (*P*_FWE_ < 0.05 and FWE <0.05 height threshold). For the LLP seed, we found an association between higher IL-6 and weaker suppression of the DMN between the LLP and a cluster including the left postcentral gyrus, left precentral gyrus and precuneus across all participants. See Table 3. No other significant associations were observed.

**Table 3:** Higher IL-6 and seed-based functional connectivity between each of the 4 DMN seed regions (medial PFC, right LP, left LP, and the PCC) and the rest of the brain.

Brain area	*x*	*y*	*z*	Voxels	Peak T	*P*-value
**Medial PFC seed and IL-6—all subjects**						
456 voxels (51%) covering 10% Precentral Gyrus Left 295 voxels (33%) covering 8% Postcentral Gyrus Left 6 voxels (1%) covering 0% Precentral Gyrus Right 130 voxels (15%) covering 0% of atlas.not-labelled	−24	−26	74	887	−4.54	<0.001 (FWE)
**L lateral parietal seed and IL-6—all subjects**						
310 voxels (49%) covering 8% Postcentral Gyrus Left 256 voxels (41%) covering 6% of Precentral Gyrus Left 63 voxels (10%) covering 1% Precuneous Cortex	−26	−30	68	629	−4.56	<0.001(FWE)
**R lateral parietal seed and IL-6—all subjects**						
No significant clusters						
**Posterior cingulate cortex seed and IL-6—all subject**s						
No significant clusters						

Given the significant IL-6-DMN associations observed in our CONN regression analyses, we further tested whether measures of CTQ-physical neglect predicted the significant IL-6-DMN associations observed. Regression analyses revealed that, after accounting for age, BMI and IQ, higher physical neglect scores explained 7.8% of the variation in the IL-6—DMN correlation coefficient for the left LP—precuneus seed (*F*(1, 208) = 5.912, *P* < 0.05).

## Discussion

### Summary of main findings

The first aim of this study was to test the hypothesis that patients with schizophrenia show increased DMN functional connectivity during a face processing task compared to healthy participants. We found that patients showed significantly increased connectivity between the left lateral parietal cortex (LLP) and the cerebellum and between the left LP and left angular gyrus compared to controls. The second aim of the study was to test the hypothesis that IL-6 predicts increased DMN functional connectivity, across the total sample. We found that IL-6 predicted increased functional connectivity between the LLP and cerebellum, LLP and precuneus, and between the mPFC and bilateral precentral gyri and left postcentral gyrus. Lastly, we aimed to investigate whether IL-6 mediated the relationship between measures of early life stress and DMN connectivity. We found that IL-6 mediated the relationship between physical neglect and connectivity between the LLP and cerebellum. Again, after accounting for age, BMI and IQ, both CTQ-physical neglect scores also significantly predicted the positive association between IL-6 and LLP-precuneus connectivity. This is the first study to our knowledge that provides evidence that both higher IL-6 and higher childhood physical neglect predicts increased DMN functional connectivity during a face processing task.

### Patients exhibit weaker suppression of the DMN compared to healthy participants

Our finding that patients exhibit increased DMN connectivity compared to controls during a task is consistent with similar recent studies as well as in accordance with the abundant literature focusing on typical and atypical DMN function from previous studies. For example, we have previously reported that patients with schizophrenia show atypically increased DMN activation compared to controls during the same face processing task [19]. The present study extends these findings of increased DMN ‘activation’ during a face processing task, by similarly demonstrating an increase in DMN ‘connectivity’ during the same task. It is also well established that patients with schizophrenia show atypically reduced functional connectivity of the DMN compared to healthy controls during rest [7, 11, 20]. The present study extends these previous ‘resting-state’ FC findings to FC ‘during tasks’, i.e., by demonstrating increased FC in patients during a face processing task, between the left LP node of the DMN and multiple other cortical areas including the precuneus. Interpreting these observations in combination with [1] our recent study in the same sample (i.e., which showed reduced FC in patients (during rest) between the same node of the DMN (i.e., LLP) and other cortical areas including the precuneus [7]), as well as in combination with [2] the extant literature on typical DMN function described previously; the results of this study demonstrate atypical state dependent FC of the DMN i.e., increased DMN FC in patients, during task performance.

Collectively, this evidence leads us to speculate that this atypical pattern observed in patients, of relatively increased DMN connectivity during the tasks, represents relatively weaker suppression of

DMN related/self-referential cognitive processing. As DMN function is strongly associated with self- referential cognition and mind wandering, this increased/weaker suppression of the DMN in patients possibly indicates that patients may struggle to be more engaged with the task at hand.

### Higher IL-6 plasma predicts weaker suppression of the DMN

The finding that higher IL-6 predicts weaker suppression of the DMN is also consistent with recent studies on the subject. The mechanism of how inflammation may affect DMN function remains unknown, but several studies have now reported associations between peripheral levels of pro- inflammatory cytokines and DMN activation and connectivity [21, 22]. For example, Bradley and colleagues (2019) showed that higher levels of several pro-inflammatory cytokines were associated with decreased activation of the bilateral precuneus during reward anticipation in a sample of 46 individuals with psychiatric symptoms, during performance of a Reward Flanker Task under fMRI [22]. Aruldass and colleagues (2021) recently showed that IL-6 was associated with lower functional connectivity between regions of the default mode network in a sample of patients with major depressive disorder, using resting state fMRI (*N* = 83) [6]. The present findings extend these studies further, both by demonstrating that IL-6 is also associated with atypically increased DMN connectivity during task performance, and by demonstrating that it was IL-6 alone which was associated with connectivity and not any of the other inflammatory markers included.

### IL-6 mediates the association between CTQ-neglect and weaker suppression of the DMN

Our finding that higher IL-6 mediates the relationship between physical neglect and the observed increase in DMN connectivity is consistent with recent research. For example, we previously reported in the same sample that higher plasma IL-6 was associated with decreased resting-state DMN connectivity, and that higher IL-6 and decreased DMN connectivity sequentially mediated the relationship between physical neglect and cognitive performance of the CANTAB emotion recognition task (ERT). The present findings extend these results by showing that IL-6 predicts a pattern of relatively *increased* DMN functional connectivity during an fMRI emotion recognition task, and that higher IL-6 mediates the relationship between physical neglect and these observed increases in DMN connectivity. Early life stress involving physical neglect appears to play an important role in the significant positive association between low grade systemic inflammation and atypical DMN observations.

This evidence leads us to speculate that the ability to suppress DMN network connectivity during performance of a cognitive task is associated with exposure to early life stress and inflammation. More specifically, the findings in this study suggest that low-grade systematic inflammation (i.e., elevated IL-6) may bridge the temporal gap between childhood neglect and failure to deactivate the DMN in adulthood. It is therefore likely that IL-6 has been elevated for years after such trauma in childhood, a theory supported by a series of longitudinal studies on prolonged proinflammatory cytokine elevation post trauma [23, 24]. As mentioned above, DMN function is strongly associated with self-referential cognition and mind wandering. In the context of our earlier study showing lower resting state DMN connectivity, this study further indicates that individuals with greater CT exposure (e.g. neglect) and associated low grade systemic inflammation (measured by IL-6) may exhibit DMN dysregulations. Weaker DMN suppression, in turn, is likely to have a negative impact on cognitive functioning [8, 11, 25, 26]. If true, DMN dysregulation and low grade systemic inflammation may represent promising targets for therapeutic intervention when aiming to treat CT’s deleterious effect on cognition.

### Limitations and future directions

There are a number of limitations in this study which should be acknowledged. The childhood trauma questionnaire (CTQ) is a well-established tool for measuring previous experiences of childhood trauma, however, it does not assess the age of onset or duration of trauma. Future studies would benefit from including such factors in the analyses. The CTQ also shares limitations with other retrospective self-report questionnaires due to recollection bias, as we’ve discussed previously [7]. However, this limitation should be considered alongside recent evidence comparing retrospective and prospective recall of CT, which found moderate agreement between these measures and that both explained a similar amount of variation in negative life outcomes [27].

The face processing task used in this study did not include a behavioural component, for example, measuring facial emotion recognition accuracy. As such, future studies using facial emotion recognition tasks that simultaneously measure behavioural performance will be useful in examining whether the cognitive state-dependent increases in DMN functional connectivity observed here are predictive of social cognitive ability and social behaviour, particularly in patients with schizophrenia.

A *post hoc* analysis of current medication dosage that was calculated (using CPZ equivalents) for all patients indicated no association with immune marker levels (plasma). A limitation here is that accounting for medication in this way does not account for variability in either duration of treatment, or changes in dose over time. However, it is noteworthy that the effects reported here were observed across patients and controls, making it unlikely that our findings could be understood purely in terms of medication effects.

## Conclusion

In conclusion, CT exposure was associated with a failure to deactivate the DMN during cognitive task performance, and the pro-inflammatory cytokine IL-6 was found to predict increased DMN connectivity, and mediate the relationship between early life physical neglect and DMN connectivity. These findings extend our previous resting-state DMN findings by demonstrating that IL-6 and early life stress is associated with atypical DMN connectivity both during rest and during performance of a face processing task. Future research examining mechanisms by which IL-6 affects DMN functional connectivity will be useful in better understanding these relationships at the cellular and molecular level, and establish whether these represent potential therapeutic targets to minimise the effects of CT on cognition.
